# Statistical Report on Expenditure of London Hospitals

**Published:** 1920-10-23

**Authors:** 


### KING EDWARD'S FUND FOR LONDON.

### Statistical Report on Expenditure of London Hospitals.

The Statistical Report of the King's Fund deals with the year in which hospital management changed from war to peace conditions, and is therefore unusually interesting. The information contained in this compilation is more easily comparable with that of the preceding year, as it refers to exactly the same number of hospitals—namely, 109; these, together with St. Bartholomew's, the Cancer, the Evelina, and possibly one or two smaller institutions, represent the whole of the hospitals of the Metropolis.

The 109 hospitals dealt with, together with the two first named, provided a total of 9,988 beds in daily occupation in 1919, as compared with 11,566 in 1918. The total number of in-patients admitted during the year was 146,585, as compared with 145,212. It will thus be seen that with a much smaller number of beds practically the same number of in-patients was treated, this being so because the military patients in the hospitals during the war showed a much longer duration of stay than do ordinary civilian cases. The report shows that both in- and out-patients were more numerous, as there were 1,318,915 out-patients attending, as against 1,203,573 in the preceding year. This result was not unexpected, as many workers were returning to civil life, and, moreover, a large number of discharged soldiers were treated in the out-patient department. The Report tells us that the total number of naval and military patients treated by the London Voluntary Hospitals during the whole period, from the outbreak of war to the end of the year 1919, amounted to 122,840.

### Huge Annual Expenditure.

The total ordinary expenditure of the hospitals included in the King's Fund tables, together with St. Bartholomew's and the Cancer hospitals, representing all charges except capital expenditure, interest on borrowed money, and contributions to Convalescent Homes, amounted to £2,137,755. The receipts from the authorities in aid of the expenditure on naval and military patients amounted to £69,716, or 3.26 per cent. of the total ordinary expenditure, the balance, as is argued by the preface to the Statistical Report—namely, £2,068,039—was met out of the normal sources of hospital revenue.

Of the 12,589 total beds available in 1919, roughly 10,000 were in average daily occupation throughout the year, 2,500, or twenty per cent., being unoccupied. This point is specially referred to in the leading article in this week's issue. In the previous year there were 13,963 beds available and 11,566 occupied.

In turning to the tables representing the cost of hospitals under different headings, it is noticeable that while some headings, such as provisions and salaries and wages, show a very heavy increase, others, notably surgery and dispensary and the expenses of management, have not increased in anything like the same proportion. For instance, in the larger general hospitals with medical schools the average expenditure per bed in nine hospitals under provisions was in 1919 £57 2s. 4d., as against £52 3s. 0d. in the previous year, while under surgery and dispensary the expenditure rose from £11 4s. 5d. per bed to £13 18s. 2d. In those without medical schools the bed rate for provisions in 1919 was £53 8s. 8d., as against £42 5s. 3d. in 1918, and under surgery and dispensary the average increased from £11 11s. 1d. to £14 13s. 0d. Under some of the other groups, particularly special hospitals, the increase under neither of these headings is so marked. Hospitals for women had an average of £46 18s. 9d. for provisions last year, while in 1918 the average was £45 18s. 11d. In this group the respective figures for surgery and dispensary were £13 9s. 3d. and £12 14s. 3d.

An indication of how useful would be some knowledge of the size of the staff employed at each hospital is given by the comparatively small increase under the heading of "Surgery and Dispensary" in most cases, this being an expense which concerns patients alone, while under all the other headings there is a much larger proportionate increase, these departments of expenditure referring to both patients and staff.

### Inconsistency of Statistics.

Another curious fact, as to which at present we can offer no explanation, is that the costliness of general hospitals with medical schools has not increased at the same rate as those without. The hospitals with medical schools have always shown a higher cost than the others, but why that cost should not proportionately increase under present conditions is not apparent. In the group of larger general hospitals with schools the average cost per bed has risen from £138 to £168, while in those without schools the increase has been from £103 to £140. We find no solution to the problem in the examination of the figures of individual hospitals. Comparing examples in the two groups, the following large differences under the separate headings are noted:—Charing Cross (182 beds): provisions, £66 19s. 9d.; surgery and dispensary, £14 9s. 4d.; domestic, £51 19s. 4d.; salaries and wages, £55 12s. 10d. Middlesex (192 beds): Provisions, £55 1s. 8d.; surgery and dispensary, £10 11s. 0d.; domestic, £58 9s. 10d.; salaries and wages, £67 18s. 6d. What is the explanation? Is it that Charing Cross has a more modern and compact building than Middlesex, and therefore costs less under salaries and wages and domestic charges such as heating, cleaning, renewals, and the like? If this is the answer, it is difficult to understand why the cost of provisions is so much higher in view of the fact that a smaller staff has to be fed.

Another example of difference in cost may be taken from the second group:—Great Northern Central (207 beds): Provisions, £63 4s. 2d. surgery and dispensary, £11 13s. 10d.; domestic,

£34 13s. 5d.; salaries and wages, £32 7s. 9d. Seamen's (208 beds): Provisions, £47 18s. 0d.; surgery and dispensary, £13 1s. 7d.; domestic, £37 2s. 10d.; salaries and wages, £36 7s. 0d. The same argument might also apply here, with the same inconclusive answer.

We do not for a moment lose the spirit of the King's Fund report in making these comparisons and observations. The figures are not intended to "show up" any hospital for extravagance. The compilers of the report know as well as we do that conditions and traditions vary largely as between institutions. The baffling result of a cursory investigation of the tables leads the conscientious administrator to look below the surface, and any close and thorough review of hospital detail cannot fail to disclose certain features which may be revised with benefit to the individual institution.

